# Pearls and Pitfalls in Pediatric Kidney Transplantation After 5 Decades

**DOI:** 10.3389/fped.2022.856630

**Published:** 2022-04-08

**Authors:** Loes Oomen, Charlotte Bootsma-Robroeks, Elisabeth Cornelissen, Liesbeth de Wall, Wout Feitz

**Affiliations:** ^1^Division of Pediatric Urology, Department of Urology, Radboudumc Amalia Children's Hospital, Nijmegen, Netherlands; ^2^Department of Pediatric Nephrology, Radboudumc Amalia Children's Hospital, Nijmegen, Netherlands; ^3^Department of Pediatrics, Pediatric Nephrology, Beatrix Children's Hospital, University of Groningen, University Medical Center Groningen, Groningen, Netherlands

**Keywords:** pediatric kidney transplantation, graft survival, pediatric urology, pediatric nephrology, immunosuppression, donor selection, lower urinary tract dysfunction

## Abstract

Worldwide, over 1,300 pediatric kidney transplantations are performed every year. Since the first transplantation in 1959, healthcare has evolved dramatically. Pre-emptive transplantations with grafts from living donors have become more common. Despite a subsequent improvement in graft survival, there are still challenges to face. This study attempts to summarize how our understanding of pediatric kidney transplantation has developed and improved since its beginnings, whilst also highlighting those areas where future research should concentrate in order to help resolve as yet unanswered questions. Existing literature was compared to our own data of 411 single-center pediatric kidney transplantations between 1968 and 2020, in order to find discrepancies and allow identification of future challenges. Important issues for future care are innovations in immunosuppressive medication, improving medication adherence, careful donor selection with regard to characteristics of both donor and recipient, improvement of surgical techniques and increased attention for lower urinary tract dysfunction and voiding behavior in all patients.

## Introduction

The first successful pediatric kidney transplantation was performed in 1959 at the University of Oregon in Portland, USA (1, 2). The field of pediatric kidney transplantation (patient age 0–18 years) has continued to evolve ever since. Whereas, pediatric kidney recipients had worse outcome compared to adults in the earlier years, today outcomes are equal.

Both patient and graft survival improved dramatically; from a 1-year patient survival of 70% in 1970 into a current 1-year patient survival of 97% and 5-year graft survival of 89% (3–6). Consequently, kidney transplantation is the first choice of treatment for children suffering from end stage kidney disease (ESKD). Nowadays, over 1,300 pediatric kidney transplantations are performed each year (285 in Europe, 1,023 in the United States) (5, 7, 8).

Multiple factors led to improved graft survival and quality of life (QOL) in pediatric kidney recipients, for example new developments in immunosuppression protocols. Infections, both bacterial and viral, used to be responsible for high morbidity and mortality in the early years of transplantations (3, 9). Subsequently, clinicians became more cautious in using immunosuppressants resulting in higher rates of rejection (4, 10). A deeper understanding of the pediatric immune system and development of targeted immunosuppressive medication contributed to improved graft survival.

Pediatric transplantation differs from adult transplantation as unique pediatric concerns related to development, growth, viral infections, congenital disorders and adherence need to be managed (11, 12). Awareness of these differences, as well as optimization of multidisciplinary pre-, peri-, and post- operative care and surgical techniques, all contributed to improvement of outcome (13, 14).

Despite this increased survival rates, we aim to further optimize care for these patients. Considering the current allograft half-life of 12 to 15-years, most pediatric kidney recipients will require a re-transplantation during their lifetime (15, 16).

Moreover, since survival has increased, future research needs to focus on the long-term effects of kidney transplantation: maintaining QOL and minimizing the side effect of immunosuppressants. Due to relatively small numbers of transplantations per center, it takes time to gain expertise.

This non-systematic review presents a summary of the current available literature. The aim was to present an overview of lessons learned during the last 5 decades of pediatric kidney transplantation and to identify unresolved fields waiting to be unraveled. Analysis of still existing lacunas in pediatric kidney transplantation care are essential to further optimize outcome.

In addition we present our own results of 411 single center pediatric kidney recipients that were transplanted in our center in the time period 1968–2020.

## Methods

A comprehensive literature search was conducted in the databases PubMed, Cochrane, EMBASE and MEDLINE for relevant English-language articles. In addition we followed citations from the primary references to relevant articles that the databases could not locate. The search was based on the following MESH-terms: pediatric kidney transplantation, donor selection, donor age, living related and unrelated kidney donation, post mortal kidney donation, prognostic factors, dialysis, pre-emptive transplantation, immunosuppressive drugs, corticosteroid withdrawal, long-term outcome/ graft survival, rejection, infections, and surgical techniques, complications, including ureteroneocystostomy. All abstracts were screened for relevant articles. Full text relevant articles were reviewed and included.

This article focused on pre-operative issues like donor selection, pre-emptive transplantation and screening for lower urinary tract dysfunction (LUTD). Besides this it covers peri-operative factors such as anastomosis technique and surgery for really small children. Eventually it describes post-operative factors like graft- and patient survival, immunosuppression and the need for transplantectomy.

In order to evaluate our own practice and to identify dissimilarities with previous research we compared outcomes retrieved from existing literature to outcomes of 411 single-center pediatric kidney recipients transplanted between 1968 and 2020 in our center. For this, population data were analyzed using SPSS Statistics 25.0 and Graphpad Prism 5.0. Differences were considered statistically significant at *p* < 0.05.

## Results

In this section we will present an overview of current literature on the most important (modifiable) factors in pediatric kidney transplantation (age 0–18 years) and highlight existing controversies that remain to be clarified.

### Survival

#### Patient Survival

Since 1959, patient survival increased significantly. Whereas, 5-years patient survival was 91% before 1990 it improved to up to 98% after 2010, mainly due to decline in infections (17). Although the number of infections decreased over time, it is still the most important cause of death in pediatric kidney recipients (28%) (18, 19).

As infection rates decreased other long-term factors became more important like malignant diseases and cardiopulmonary complications. Both these complications are responsible for respectively 12 and 15% of current 5- and 10-year patient survival (18).

#### Graft Survival

In 1990, 5-year graft survival was ~77% for the living donations (LD) and 57% for the deceased donations (DD) (20). In this period, rejection rates were as high as 80–90% and rejection (both acute and chronic) was the major cause of graft loss (4).

Nowadays, acute rejection rates have decreased to 10–15%, due to improvements in pre-operative donor selection, peri-operative management and immunosuppressive regimes. Although rates of acute rejection have immensely diminished, chronic rejection and acute rejection remain the leading causes of graft loss (21 and 15%, respectively) (17). Other important causes of graft loss are disease recurrence (10%) and vascular thrombosis (11%).

#### Radboudumc Amalia Children's Hospital

In our center, since 1968, 411 kidney transplantations have been performed in patient aging 0–18 years.

Similar to the literature, patient survival increased from a 5-year survival of 93% in recipients transplanted before 1990 to 98% when transplanted after 2010. Overall, infection was the most important cause of mortality (25%), followed by cardiovascular complications and malignancy (17 and 14%, respectively).

**Figure 1:**
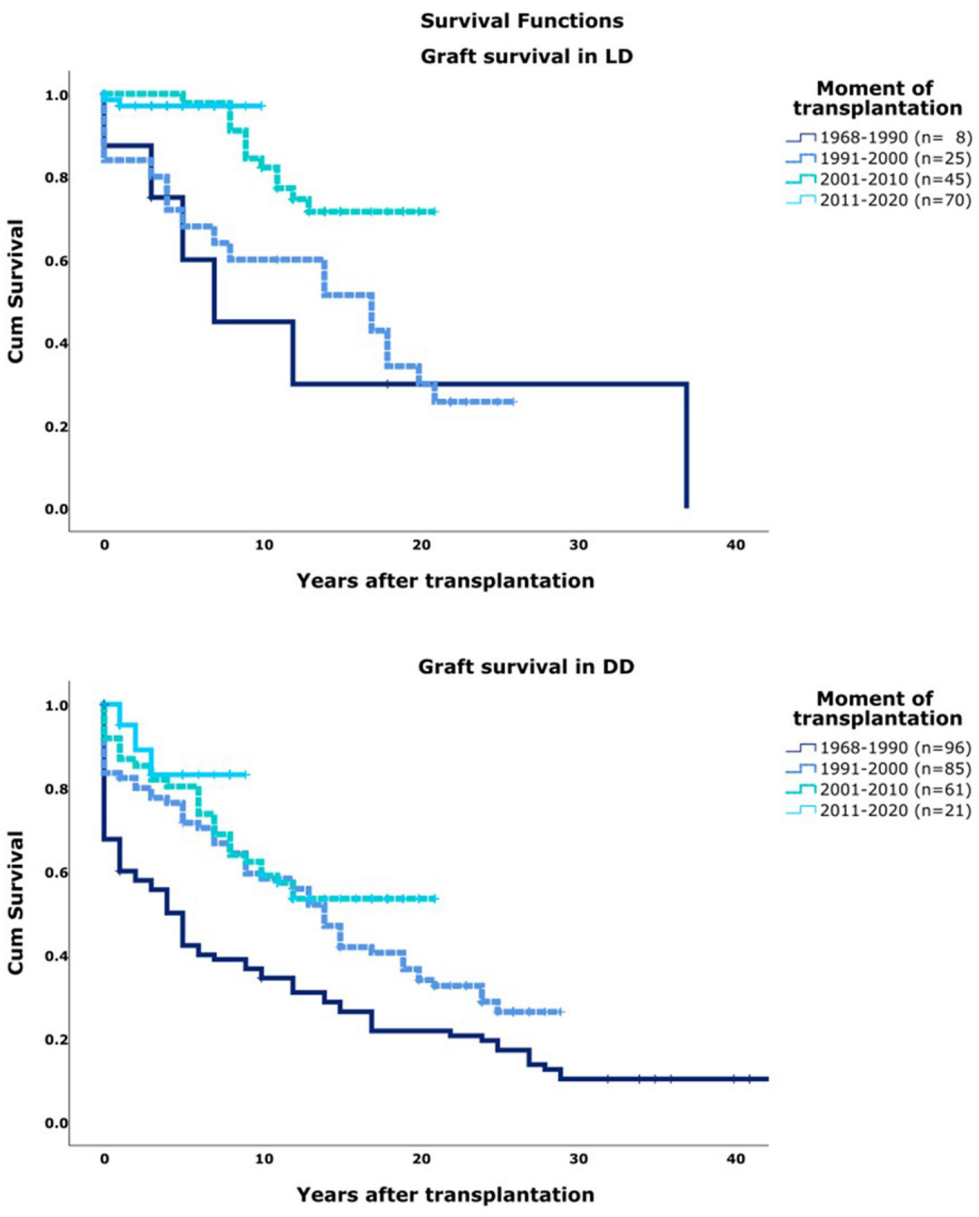
Graft survival per decade of transplantation. T0, moment of transplantation. DD, deceased donor; LD, Living Donor.

Graft survival increased significantly over ascending era's even when stratified for DD/LD [Figure 1 (*p* < 0.01)]. In general, LD resulted in better 5, 10, and 20 year graft survival compared to DD (*p* < 0.01). Majority of graft loss in our center was caused by both forms of rejection (75%). Other important causes were recurrence of primary disease (5%) and thrombosis (6%). Causes of graft loss did not change over time.

### Pre-transplantation Dialysis vs. Pre-emptive Transplantation

Most pediatric kidney recipients are exposed to dialysis prior to their transplantation [51% to hemodialysis (HD) and 28% to peritoneal dialysis (PD)]. Current incidence of pre-emptive kidney transplantation (PKT) in children is 20% in Europe (7). However, rates of PKT vary greatly between countries with 2% PKT in Italy, 41% in the Netherlands and 61% in Norway (7, 21). This wide range might be partly due to differences in local allocation policies as these vary among countries (7). In adults, PKT was shown to be superior to post-dialysis transplantation as it results in favorable graft and patient outcome as well as improved QOL (22, 23). However, this is more controversial in pediatric patients.

#### PKT vs. Dialysis

In theory, dialysis has several disadvantages for children suffering from ESKD. Dialysis is associated with negative effects on growth, anemia, bone mineral regulation and cardiovascular status due to chronic volume overload and uremic toxins (24–26). Moreover, surgery for dialysis access makes patients more prone to infectious complications and avoiding dialysis might preserve the vessels for the future and increase graft survival (27).

Despite these theoretical objections to dialysis, literature showed conflicting results of PKT in children. Some studies report better graft and patient survival in PKT (28–30) whilst others found similar results for both pre-emptive and post-dialysis transplantation (31–33). However, some studies were performed after DD, others after LD and some after both. It should be noted that most of these studies had limited follow-up time.

There are several possible explanations for these conflicting results.

Undervaluation of PKT might be due to the relatively short duration of dialysis in children compared to adults. Period of dialysis is thought to predict survival since a longer time on dialysis was associated with increased risk of adverse events (23, 26, 34). In adults, average time on dialysis before transplantation is 5 years, whereas for children this is <1 year (5).

Amaral et al. showed significant graft survival benefits in pediatric recipients after PKT compared to those on dialysis for as little as 6 months (28). This was confirmed in a large adult cohort by Prezelin et al. (23) and advocates PKT regardless of the duration of dialysis.

Overvaluation of PKT might be caused by selection bias. Recipients of PKT are more likely to be healthier, better nourished, have better residual kidney function and more likely to receive a graft from a LD compared to those on dialysis (22, 35).

Another factor is lead time bias. The treatment of choice, in this case transplantation, was given at an earlier stage in PKT than after dialysis which results in a longer follow-up after transplantation. This can cause a perceived advantage in PKT, as the graft survival time is calculated from an earlier starting point than in post-dialysis transplantation (36).

Additionally, a possible explanation for the conflicting results is the limited follow-up time. As the risks of dialysis are mainly cardiovascular disease, consequences are expected later in life and affect patient survival rather than graft survival. More long-term research could provide better answers.

#### Promoting PKT

An important barrier to PKT is a lack of patient education as many patients and potential living donors are not aware of the possibilities of PKT (37, 38). Besides, it remains difficult for patients to address the topic of living donation with their loved ones (39, 40). In the Netherlands, a home-based educational program was introduced to increase knowledge and improve communication among patients who are yet to undergo renal replacement therapy (41). This resulted in increased rates of PKT, probably because of the involvement of patients social network to the program (42, 43).

#### Radboudumc Amalia Children's Hospital

**Figure 2:**
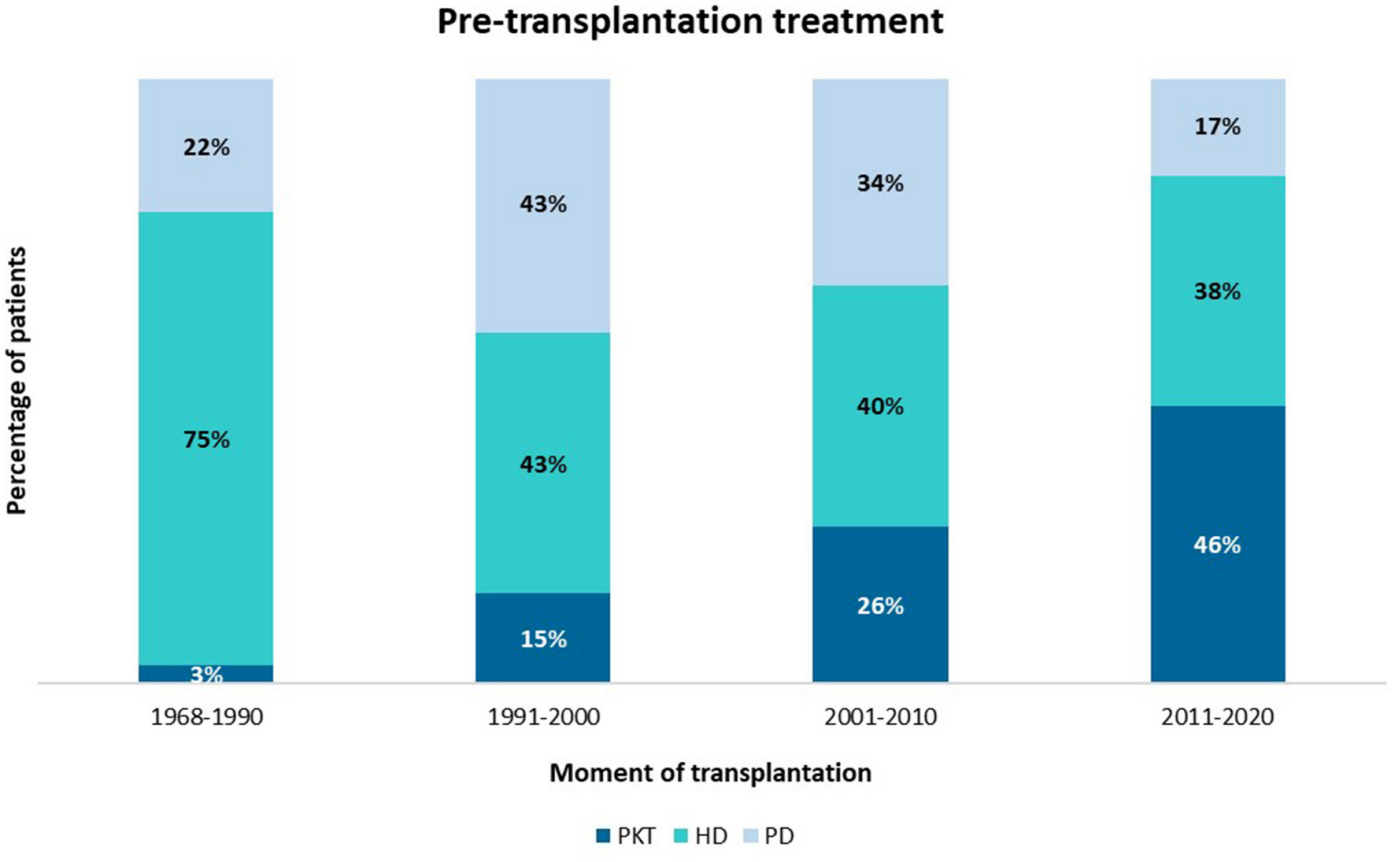
Mode of pre-transplantation treatment per era of transplantation. PKT, pre-emptive kidney transplantation; HD, hemodialysis; PD. peritoneal dialysis.

In our center, the rate of PKT increased over time, from 6% before 1990 to a current number of 46% (Figure 2). Median time on dialysis was 15 months [IQR 9–32], the majority of patients (47%) were treated with HD vs. 28% with PD. When corrected for decade of transplantation, neither pre-transplantation treatment nor duration of dialysis significantly affected graft survival or patient survival.

### Donor and Recipient Selection

#### Donor Selection

##### Deceased Donors (DD)

Donor selection is an important factor for graft survival: LD is considered to be preferable to DD since it results in both better graft and recipient survival (18, 44). However, there is a disparity between supply and demand for grafts which necessitates DD. Besides, access to kidney transplantation and allocation procedures vary widely among countries. In most European countries, pediatric patients on a transplantation waiting list are given priority, which might have resulted in increased allocation of young DD kidneys to pediatric patients (21).

##### Living Related Donors (LRD)

Living related donation allows proper HLA-matching and limitation of ischemia time. And using living (un)related donors allows paired exchange.

In pediatric transplantation, LRD rates exceed those in the adult population since the donors are often the parents of the child.

Previous studies stated that maternal donation might be preferable to paternal donation since it results in decreased rate of acute rejection in the youngest recipients (<4 years) (45, 46). This phenomenon could be caused by microchimerism which is defined as the persistent presence of maternal cells in organs of the child due to bidirectional transfer of cells trough the placenta antenatally (47). However, this effect remains controversial as other studies found a negative association between graft survival and maternal donation. They stated that paternal grafts result in better long-term outcome because of the increased size and amount of nephrons of male kidneys (48–50).

Despite an overall increase in LD in Europe, the numbers of patients on donor kidney waiting lists are stabilizing (51). Impediments to find (living) donors include concerns on blood group incompatibility, donor age and health of the donor.

##### Living Unrelated Donors (LURD)

A possibility to expand the donor pool is the use of living unrelated donors. Graft outcome after LURD was shown to be superior to DD (52–54). In adults, rates of LURD are relatively high since donors are often the partner or a friend.

Although ethics of organ donation have always been a sensitive issue, this might be of more importance in (unrelated) living donation (4). Several countries in the Middle East prohibit LURD in order to avoid organ trafficking (55). On the other hand, the Iranian government operates a paid LURD kidney transplantation program also known as the Iranian model (56).

Non-commercial LURD is allowed in most Western Countries such as the Netherlands, following the recommendations of the Council of Europe (57).

##### Donor Age

Previous research in adults has shown advanced donor age to result in poor graft survival (58, 59).

This deleterious effect of high donor age seems less evident in pediatric recipients. Chesnaye et al. showed that the risk of graft failure in older living donors (50–75 years old) was similar to that of younger living donors (60). On the contrary, Trnka et al. showed that an increasing age difference between donor and recipient was associated with decreased graft survival (61). Allowing healthy elderly to donate their kidney remains debatable, however it might benefit against graft shortage.

##### HLA-(mis)match

Conflicting results have been published concerning the effect of HLA-matching. Whereas, some studies showed superior results for children receiving a poorly HLA-matched LD kidney compared to a well-matched DD kidney (62, 63) most studies showed the exact opposite (61, 64–66).

However, the definitions of poor and well-matched donation differ between studies. Additionally, geography might play a role in this context as cold ischemia times would be increased if DD grafts need to travel large distances.

Currently, the trade-off between time on a waiting list and HLA-mismatching remains unsolved and needs further exploration in the future.

##### Radboudumc Amalia Children's Hospital

**Figure 3:**
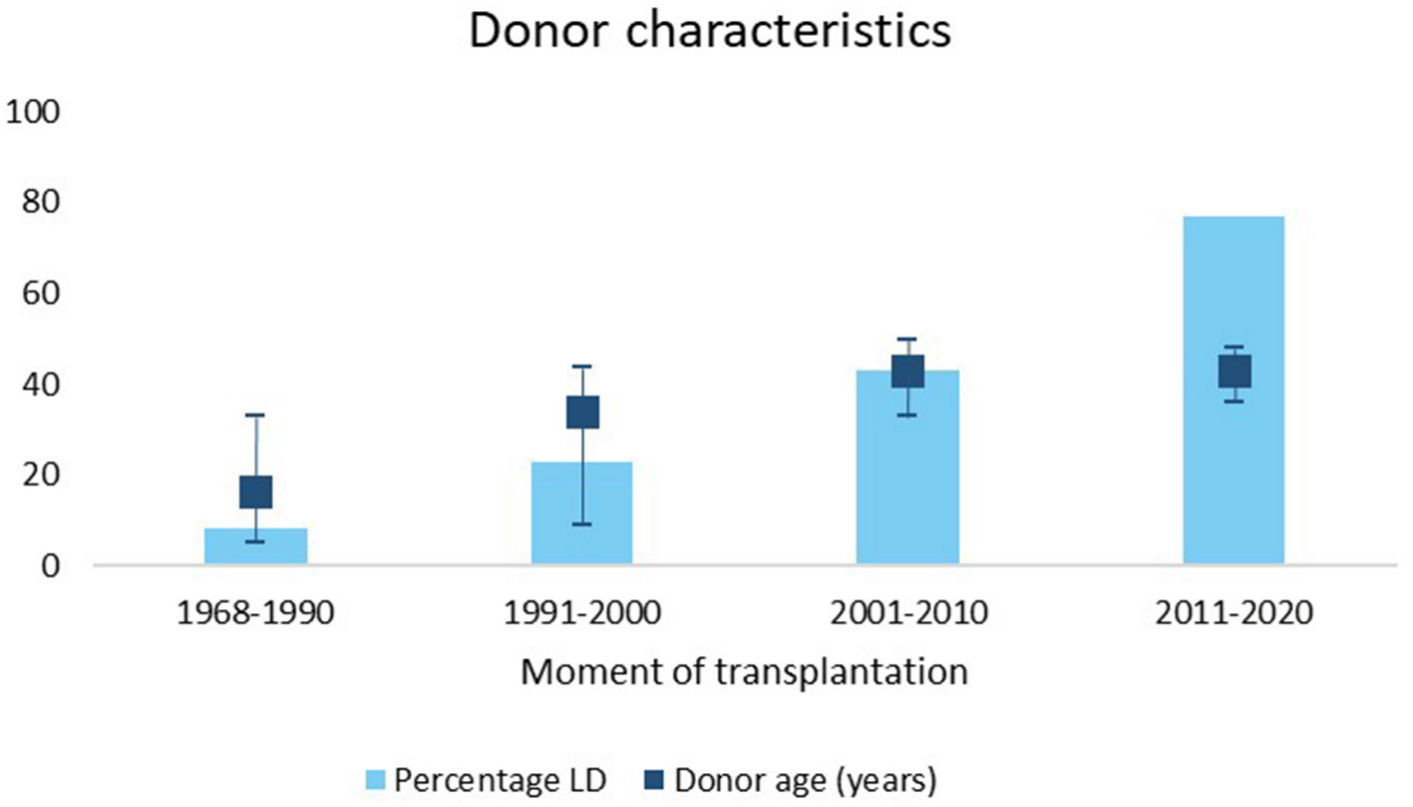
Percentage of living donors and IQR donor age stratified for different time periods. LD, living donor.

In our center, the rate of LD and the donor age increased over time (Figure 3). Median graft survival after LD was longer compared to DD with a median survival of 20 years (95% CI 16–24) vs. 12 (95% CI 9–15) (*p* = 0.01) even when stratified for decade of transplantation.

In total, 3% of the kidneys were from a LURD and 33% from a LRD, mostly parents (18% father and 11% mothers). There was no significant difference in graft survival between maternal and paternal donors.

In addition, median number of HLA mismatches significantly increased over time (*p* < 0.01).

#### Kidney Transplantation in Small Children

Kidney transplantation in children under the age of 1-year is rare (0.5–5% of all pediatric kidney transplantations) and poses surgical challenges in terms of size differences in body cavity and vessel diameters (67, 68). Previous studies use various definitions of “very small children” ranging from younger than 1 or 2 years of age to a weight below 10, 15, or 20 kg (69–73).

##### Special Considerations in Small Children

Trying to fit an adult-size graft in a small retroperitoneal space is challenging and might lead to increased abdominal pressure and impaired graft vascularization (74, 75). In addition, a relatively large graft demands an increase in renal blood flow which asks for aggressive fluid management in order to optimize renal perfusion (70, 76–78).

In adults, the graft is commonly placed retroperitoneal whereas in children graft placement depends on the size of both the abdomen and the graft. In the youngest children (generally <15 kg), many centers use intra-abdominal placement and implant the graft on the inferior vena cava and aorta rather than on the more commonly used iliac vessels (14, 72, 79, 80).

Despite the advantage of larger space for the graft, intra-abdominal placement has some disadvantages including risk on bowel injury and a more difficult access for future interventions such as graft biopsies or PD.

Previous studies showed small children (under 15 kg) to be at increased risk for thrombosis compared to older children (OR 0.11–0.85) (71, 81, 82).

Additionally, very specific individual management might be needed in case of rare and complex associated medical conditions associated with prematurity, severe anatomical anomalies, mental illnesses, syndromal anomalies or extensive urological, or surgical previous procedures. This care should include a medical point of view of all related specialties, nursing care experts and psychological support.

##### Innovations

Despite the relatively high complication rates, graft- and patient survival in small children have improved in the last decades (1-year graft survival of 50% 1978–2000 vs. 97% 2000–2016). Nowadays, outcomes of transplantation in infants are comparable to those in older children (71, 73, 77, 78, 83). These improvements might be due to the high rates of living donations, the operationalization of dedicated multidisciplinary teams and improved immunosuppressants and diagnostics.

Accurate imaging of recipients and potential donors allows health care providers to make well-educated choices regarding the favorable surgical technique and postoperative care.

##### Timing

Although kidney transplantation is shown to be safe and successful in very small children, there is still some controversy concerning the optimal timing of the transplantation. On the one hand, early transplantation could avoid dialysis and allows better physical and neurological growth in these young children. On the other hand, early exposure to immunosuppressive therapy might result in severe infections and long-term side effects. Besides, with current graft survival, transplantation at a very young age implicates multiple re-transplantations in a life time, which are known for decreased graft survival compared to primary transplantations (84–86).

Currently, there is no consensus on a minimal age or weight for transplantation. Whereas, some centers perform transplantations in children above 6 kg, others use a minimum age of 2 years (69, 72, 87). Further work is required to determine optimal timing with regard to long term outcome.

##### Radboudumc Amalia Children's Hospital

In our center, we started a program for transplantations in children weighting <20 kg in 2012. A multidisciplinary team including pediatric nephrologists, pediatric urologists, pediatric anesthesiologists, pediatric ICU specialists, vascular surgeons and pediatric surgeons as well as a paramedical team started this program with satisfying outcome (77).

Special attention is paid to the disparity between the size of the kidney graft and the length of the recipient and therefor the size of the body cavity.

Since the start of this special program in 2012, 13 children with a weight below 15 kg have been transplanted thus far (mean age 3.4 ± 1.6 years, mean weight 12.7 ± 1.5 kg). Up to this moment all recipients are alive with a functioning graft [median follow-up of 89 months (range 3–221)].

In contrast to other studies, we didn't find a difference in graft survival between age groups (*p* = 0.26) (Appendix).

### Urological Work-Up and Follow-Up

Urological causes for ESKD are seen in 25–40% of the pediatric kidney recipients and encompass mainly posterior urethral valves (PUV), vesico-ureteral reflux (VUR) and neurogenic bladder (88). Nephrological causes include renal dysplasia, hereditary kidney diseases such as ciliopathies, focal segmental glomerulosclerosis and other types of chronic glomerulonephritis (7, 89).

Lower urinary tract dysfunction (LUTD) might affect pediatric kidney graft outcome (90). LUTD is an umbrella term that includes several urological items reflecting the function of the bladder and lower urinary tract. Exact definitions of LUTD vary widely across the literature which makes comparison of research data on LUTD challenging. Whereas, some diagnose LUTD using uroflowmetry and frequency voiding charts others include all children with for example a bladder augmentation and intermittent catherization without other diagnostics. By consequence, there is little consistency on the prevalence of LUTD (91, 92). Although LUTD was particularly thought to be a problem in urological patients, LUTD was found to be fairly common in all kidney recipients despite underlying cause for ESKD (93–96).

In children with PUV, myogenic changes in the bladder wall result in abnormal contractility and different sensations in for example a full bladder. This ultimately might induce abnormal voiding behavior and high intra-vesical pressures. The normal bladder cycle is interrupted in patients with PUV and high pressure might cause fibrosis in the bladder walls. The prevalence and severity depends on the severity of previous mentioned changes in the bladder wall (97–99). Therefore, long-term graft survival might be worse in recipients with PUV than in those with other forms of congenital anomalies (100).

There are several factors that could contribute to development of LUTD in pediatric renal recipients without an urological history. Long pre-transplantation polyuria may lead to overdistension of the bladder which results in diminished sensation and therefore abnormal voiding patterns (101). On the other hand, oliguria results in low-capacity bladders. These low-capacity bladders cannot adjust to a sudden increase of urine volume, e.g., after transplantation. This might result in high intra-vesical pressures that lead to LUTD and a subsequent deteriorating effect on graft function (93, 95, 96).

At this moment, little information is available on the effect of immunosuppressive medication on the bladder wall (102).

The general believe is to treat LUTD as much as possible before transplantation in order to protect the kidney graft from a high-pressure lower urinary tract (99). Although the occurrence of LUTD is associated with the risk on urinary tract infections (UTI), the effect on graft survival and graft function remains less clear (103–105). Some studies showed LUTD to negatively influence graft survival whereas the majority of previous research did not (90, 106, 107). As definitions of LUTD vary, it remains difficult to draw general conclusions from these studies.

Regardless of the actual effect on graft function, LUTD in children results in diminished quality of life and increased morbidity, especially in the case of urinary incontinence (108–110).

#### Screening for LUTD

Optimal timing and screening methods remain to be determined. Multiple diagnostic tools are available including (digital) frequency voiding charts (FVC), Urodynamic studies, Ultrasound (US), Voiding Cystourethrogram (VCUG), uroflowmetry (FR), and post voiding residual (PVR) measurement. Since most patients do not need invasive diagnostics, PVR was found to be the most accurate predictor for prognosis (111). However, a combination of multiple non-invasive diagnostics might be favorable to get a good understanding of voiding behavior and bladder function. In patients with PUV or neurogenic bladder a VCUG and urodynamic studies prior to transplantation are recommended (99, 112).

Urotherapy, which is defined as non-surgical, non-pharmacological treatment for LUTD, is considered to be the cornerstone in the treatment in otherwise healthy children (98, 113). It encompasses education, behavioral modification, registration of voiding habits and life-style advice (113). In this light, it seems reasonable to not only screen all children before kidney transplantation, but also start with urotherapy to allow earlier intervention. On the long term this might lead to subsequent decline in morbidity and better graft outcome.

#### Interventions

The timing of bladder augmentation remains controversial. If indicated, bladder augmentation is mostly performed before transplantation (114). An advantage of this timing is that the bladder can heal before starting immunosuppressive medication. Whereas, multiple studies show that pre-transplantation bladder augmentation is favorable over post-transplantation augmentation (115, 116), others reported equal outcome in children that were transplanted first (117). Arguments against pre-transplantation augmentation include the increased risk of infection and the scenario of a dry augmentation. In addition, peritoneal dialysis might be a relative contra-indication (114, 118). Augmentation concurrent with transplantation was discouraged by most authors because of the increased risk on surgical complications (118, 119).

The need of pre-transplantation bladder cycling remains unclear, although several authors argument that this would improve outcome, it was not shown to be beneficial (120, 121).

#### Asymptomatic Bacteriuria

Another controversial topic is the treatment of asymptomatic bacteriuria (AB) which occurs in 17–51% of adult kidney recipients (122). Multiple studies among which a recent Cochrane review stated there is insufficient evidence for treating asymptomatic bacteriuria with antibiotics, especially in the light of possible resistance (123–125). None of the included studies showed significant effects of antibiotic treatment on graft survival or graft function. Therefore, one can doubt if screening for asymptomatic bacteriuria is useful in this population.

The role of bladder rinsing with hyaluronic acid and chondroitin sulfate is unclear. Several studies have shown beneficial effects in individual pediatric patients, but those were limited in the number of participants (126, 127).

#### Radboudumc Amalia Children's Hospital

In our center 22% (*n* = 89) of the 411 pediatric kidney recipients had an urological cause for their ESKD. In a prospective study of 56 patients, we screened all recipients for LUTD and treated them if indicated. LUTD was diagnosed in the majority of patients (71%) regardless of the underlying cause of kidney failure. This indicates that most pediatric transplant recipients do not have adequate voiding behavior, normal bladder capacity, and micturition frequency.

### Anesthetic Issues

Peri-operative care for pediatric kidney recipient differs from adult care and because of the rarity of pediatric kidney transplantation, there are no evidence-based guidelines available. There are multiple issues requiring special attention in this population.

#### Anesthesia Technique

Kidney transplantation requires general anesthesia, endotracheal intubation and controlled ventilation. There are various sedatives used, however no specific drugs were shown to be preferable. Sevoflurane might have a beneficial effect on hemodynamics although concerns have been raised about its nephrotoxicity (128, 129).

Many patients with ESKD have impaired long function because of fluid overload and leakage of alveolar membranes. Therefore, lung protective mechanical ventilation might be beneficial in pediatric kidney transplantation (130, 131).

#### Hemodynamic Challenges

One of the major challenges during kidney transplantation is the preservation of adequate graft perfusion. Although a minimum mean arterial pressure (MAP) of 70 mmHg is recommended in adults, administration of excessive fluids or vasopressors might be harmful in children. Therefore, adjustment of the target MAP to the donors MAP and visual judgement of perfusion is favored in this population.

Methods for managing hemodynamics are the administration of fluids and the use of vasoactive medication.

Norepinephrine is recommended in patients that do not respond to fluid administration. It prevents post reperfusion hypotension which is commonly seen in small pediatric patients that receive a kidney form an adult donor (76, 131).

In very young children (<5 years) hemodynamic challenges are even bigger. Because of large differences in vascular sizes between donor and recipient, renal arterial blood flow can be compromised. Additionally, a large kidney demands a persistent increase in cardiac output of the child in order to meet the flow demands of the graft. Therefore, close hemodynamic monitoring is of utmost importance in these patients and cardiac output measurement during anesthesia should be considered (76).

#### Radboudumc Amalia Children's Hospital

In our center, anesthesia for children below 40 kg is done by dedicated pediatric anesthesiologists.

During surgery, multiple monitoring tools are used including end tidal CO_2_ and oximetry. In children <20 kg we use Pulse Contour Cardiac Output (PiCCO) technique for advanced hemodynamic monitoring (131).

Of 411 transplants 4 had primary non-function. Median duration of cold ischemia was 16 h [IQR 2–26], median duration of second warm ischemia was 35 min [IQR 38–42].

### Surgical Issues

#### Nephrectomy

Native kidneys are removed before transplantation if they are expected to be of short- or long-term risks to the kidney recipient or the graft. Indications for nephrectomy include high risk of recurrence of native disease (e.g., nephrotic syndrome, focal glomerular disease), congenital anomalies, chronic infection, refractory hypertension, and malignancy (132).

However, some of these indications are rather relative indications. Arguments against this procedure include the need of additional surgery and anesthetics, the risk of peritoneal laceration and the benefits of residual urine production.

Theptimal timing of nephrectomy has to be determined as well. Whereas, nephrectomy was commonly conducted before transplantation, some authors are in favor for post-transplantation nephrectomy because of the benefits of pre-emptive transplantation, minimization of sensitization and better clinical condition (133). Other studies showed favorable outcome for simultaneous transplantation, as this limit the amount of operations and anesthesia (134–136).

Currently, nephrectomy by means of a surgical intervention is the most common form of practice. However, various studies reported on alternatives like renal arterial embolization and medical nephrectomy by means of indomethacin or an ACE inhibitor (137–140). Although effectiveness of embolization was shown to be higher compared to medical nephrectomy, side effects like hemorrhage, postembolization syndrome, or non-target embolization were more severe.

However, only studies with limited patients were eligible, therefore future research should focus on these less invasive methods.

##### Radboudumc Amalia Children's Hospital

In the 100 most recent patients in our center, pre-transplantation nephrectomy was performed in 21%. Common indications were steroid-resistant nephrotic syndrome (38%) and large polycystic kidney volume (14%).

We conducted a successful medical nephrectomy using ACE inhibitor in 6 out of 8 patients with nephrotic syndrome (139).

#### Surgical Complications

##### Urological Complications

Urological complications after kidney transplantation can be divided in early (4%) and late complications (9%). Urinary leakage (2%) and early ureteral stenosis (1%) (due to limited ureteral perfusion) or lymphocele needing drainage occur in the 1st month after transplantations, whereas late complications are mostly UTI (15–58%) and late ureteral obstruction (5–8%) (75, 90, 124, 141–145). The latter is often the result of fibrosis, infection or rejection and therefore distinct from early stenosis (146).

The placement of a temporary ureteral stent remains controversial. Previous literature showed that stenting was associated with increased risk on BK viremia and UTIs (147, 148). This might be caused by the mechanical trauma induced by stent placement which activates latent BK virus (148). However, other studies showed ureteral stenting to be protective against urological complications such as stenosis or leakage (149, 150). In adults stents are commonly used and associated with a reduction in urological complications from 7 to 1.5% (151). Further work is required to determine the trade-offs between the positive effects of stents preventing post-operative complications and the negative effect of increased risk of BK nephropathy.

In adults, JJ stenting was shown to be preferable to percutaneous stents in terms of recovery, however duration of drainage remains debatable (152).

A Cochrane Review in the adult population showed that early removal (<15 days) of bladder indwelling and per-urethral stents might decrease the risk on UTI, however differences are small (153). The benefits of stenting in the pediatric populations remain unclear, currently many centers chose early stent removal in order to prevent urological complications and limit the number of infectious events (72, 149). The use of suprapubic bladder catheter or transurethral catheter is also based on individual preference and specific patient characteristics.

Additional clinical trials are needed to support this practice.

##### Vascular Complications

Vascular complications are an important cause for early graft loss and include mainly renal thrombosis (3–12%) and arterial stenosis (3–15%) (149, 154, 155).

Venous thrombosis is considered the most common cause of early graft loss and especially small children are at risk for developing thrombotic complications (73, 156). However, the benefits of anticoagulation should be balanced upon the risk on hemorrhage and practice differs between centers (75, 157, 158). Studies on thrombotic prophylaxis showed a reduction of thrombotic events for anticoagulant use, however because of the poor quality of the data and the diverse protocols no solid conclusion can be drawn (159).

Renal artery stenosis is associated with hypertension and progressive graft dysfunction and can be due to kinking or trauma of the artery, vascular rejection, inadequate suturing or atherosclerosis.

##### Lymphatic Complications

The most important lymphatic complication after transplantation is lymphocele, which occurs in 0.5–22% of the recipients (149, 160–162). It is caused by transection of lymphatic vessels of either donor or recipient and develops usually in the 1st week after transplantation.

Lymphocele can result in compression of the graft vessels, ureter or bladder outlet and therefore cause decreased graft function. Analysis of the aspired fluid can differentiate from hematoma, urinoma and seroma. In pediatric recipients, a higher age, BMI and number of transplantations were associated with the development of lymphoceles (160). In addition, several studies showed that sirolimus may is correlated with lymphocele formation (163–165).

##### Radboudumc Amalia Children's Hospital

According to our protocol, all children have a ureteral splint for 5 days in patients older than 4 years and 7 days in patients <4 years and a transurethral or suprapubic catheter for 7 and 9 days, respectively. Protocol antithrombotic prophylaxis for patients older than 12 years exists of daily 2500 IE dalteparine post-operative until good mobilization. Specific anticoagulants such as Direct Oral Anticoagulants (DOACs) or Vitamin K antagonists are given on indication. We don't use prophylaxis for arterial thrombosis. In total, 13 patients (3%) lost their graft due to thrombosis, all before 2005.

#### Ureteral-Bladder Anastomosis

The ureteroneocystostomy (UNC) technique is one of the surgical factors that might influence the urological complication rate (166).

##### UNC Methods

The method used for the neo-ureteral-bladder anastomosis has changed over the years in the adult population. Where an intra-vesical (anti-reflux) technique was common in the past, nowadays this has changed to an extra-vesical approach with or without anti-reflux technique (166).

**Figure 4:**
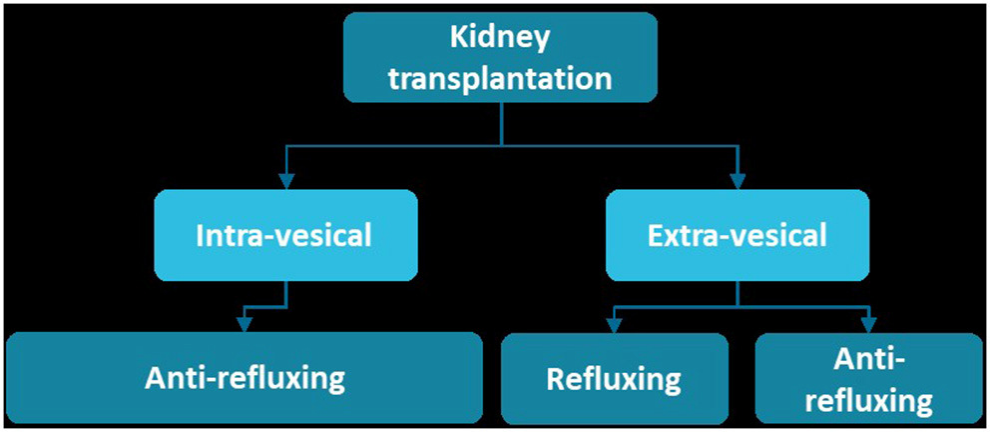
Overview of different ureteroneocystostomy methods.

Overall, UNC techniques can be divided in being either intra-vesical or extra-vesical and refluxing or anti-refluxing (Figure 4).

With the intra-vesical Leadbetter-Politano (PL) technique an anti-refluxing tunnel is created to prevent vesico-ureteral reflux (VUR) (167). This technique was originally performed in most transplantations and requires 2 cystostomies (166).

After some time the easier extra-vesical modified Lich-Gregoir (LG) technique gained more popularity. In the LG technique, a single cystotomy is performed and the distal ureter anastomosis to the bladder is covered by detrusor muscle with the intention to create a valve effect and prevent VUR (168). Over time, new methods were reported such as the “U-stitch” technique and the “full-thickness” technique. The latter is an extra-vesical refluxing technique in which the ureter is anastomosed to the bladder without coverage of detrusor (151, 166, 169, 170). Earlier research showed favorable outcome for the LG technique in terms of urinary leakage and hematuria compared to PL and U-stitch methods (169, 171).

Currently, the “full thickness” method is commonly used in the adult population which has no anti-reflux mechanism (151). This technique minimalizes any risk for ureteral obstruction with comparable outcomes as anti-refluxing techniques (172).

##### UNC in Pediatric Patients

Little is known about the optimal UNC technique in the pediatric population. Although an anti-reflux technique might be favorable regarding the increased risk of VUR in pediatric recipients (173), it could increase the risk for ureteral obstruction.

The importance of VUR as a complication after kidney transplantation is debatable since it is often asymptomatic and might not influence long-term outcome in adults (174, 175). The exact prevalence of VUR in children is unknown since routine VCUG after transplantation is not standard practice in most centers. Besides, the fact that a part of this population is not continent yet makes it difficult to compare to adult care. Whereas, Ranchin et al. showed VUR incidence up to 58% despite anti-refluxing methods (176), symptomatic VUR occurred in only 5–12% of the pediatric kidney recipients (177–179). Post-transplant obstruction was reported in 8% of cases (141, 142) and post-transplantation UTI in 15–58% (180–182).

Altogether, the choice of UNC technique in pediatric kidney recipients remains difficult and the long-term effects are still unknown. It would be worthwhile to compare outcomes of anti-reflux vs. reflux techniques in pediatric kidney recipients specifically.

##### Radboudumc Amalia Children's Hospital

In our center, from 2000 all anastomoses were created using a refluxing extra-vesical technique. In 100 recipients that received their graft between 2002 and 2018 55% had least one UTI, 20% had recurrent UTI's. VCUG was done in 11 symptomatic children of which five were diagnosed with VUR.

#### Transplantectomy

As stated before, graft failure is mainly the result of rejection or thrombosis. When a graft fails, one has to choose between either leaving the graft *in situ* or performing a graft nephrectomy (GN).

It is well–established that GN is indicated in cases of vascular thrombosis, hyperacute rejection, and therapy resistant malignancy (183–187). Relative indications include severe graft pyelonephritis, the wish of withdrawal of immunosuppressants and symptoms of the intolerance syndrome (185, 188, 189). The removal of an asymptomatic non-functioning graft remains controversial.

In adults, GN is performed in about 35% of patients with a failing graft (187, 190). In pediatric recipients over 50% of patients with a failing graft had GN, although data on the pediatric population are scarce (186, 191). This difference might be caused by the higher rates of acute rejection in children, which was thought to be caused by a more vigorous immune response in children (192). In both adults and children, recipients were most likely to have GN when graft failure occurred in the 1st year after transplantation (185–187, 191).

Considered benefits of GN include reduction of inflammation, discontinuation of immunosuppression and possible reduction of the number of donor specific antibodies. However, surgery for graft removal may cause considerable peri- and post-operative morbidity such as inflammation and hemorrhage. Post-operative mortality rates ranged from 1 to 39% and was mostly caused by sepsis (17%). Moreover, re-transplantation outcomes are worse after GN compared to no GN (187, 193, 194).

Moreover, GN is associated with higher donor specific antibodies because of the potential absorptive capacity of the graft (195). Previous studies showed a longer interval between graft loss and re-transplantation after GN (186, 187, 193). Moreover, minimal residual urine and erythropoietin production from the failed graft may be preserved when immunosuppression was continued (196).

##### Allosensitization After GN

To establish the impact of GN on both allosensitization and graft outcome remains challenging because of multiple confounding factors. However, timing of GN is thought to be an important factor. Sener et al. showed that patients that had GN in the 1st month after transplantation had lower panel reactive antibodies (PRA) and reduced risk on future graft failure compared to those who did not have GN (197).

In contrast, patients who had late GN (>1-year) were at increased risk for future graft failure and had increased PRA (187, 197, 198). Wang et al. reported no difference in patient and graft survival between those who underwent GN and those who did not, whereas Ayus et al. showed a 32% lower risk on morbidity after GN (190, 193).

The (dis)continuation of immunosuppressants after GN remains debatable, since continuation would prevent allosensitization while increasing the risk on infections, vascular disease and malignancy (144, 199). In children, considerations might be different than in adults since they are more likely to have a re-transplantation.

There is only limited research on GN in children. Whereas, high rates of morbidity and mortality were seen in adults, outcome in pediatric GN was shown to be good. No major complications, re-operations and blood transfusions were reported in the few studies on pediatric recipients (186, 191).

##### Alternatives

Renal artery embolization (RAE) was thought to be a minimal invasive alternative to GN as it results in less surgical complications. However, the risk on necrotic pyelonephritis and post-embolization syndrome are increased after RAE and RAE as monotherapy is not widely used (200). However, using RAE as neo-adjuvant intervention before GN was shown to reduce both blood loss and operating time (201–203).

##### Radboudumc Amalia Children's Hospital

Between 1977 and 2020, 379 transplantations were performed in our center. Graft failure occurred in 108 grafts so far of which 66 (53%) were removed. There was no operative mortality and 32% of the surgeries resulted in complications which were all resolved (191).

### Acute Rejection

#### Graft Biopsies

Subclinical acute rejection was shown to be a cause for deterioration of graft function which implies that early diagnosis and treatment is favorable. Since subclinical rejection can only be diagnosed by means of graft biopsy, the practice of “protocol” or so called “surveillance” graft biopsies is under debate (204, 205). Although this allows early detection of rejection and tubular atrophy, the effect on long term graft survival remains unclear. Most studies showed comparable short term results in both patients that had protocol biopsies and those that had biopsies on indication of clinical symptoms (206). However, a recent prospective study revealed children who underwent protocol biopsies to have better renal function on the long term than the control group (205). Despite the prospective nature of this study, there are some limitations such as different immunosuppressive regimens and the lack of randomization.

Literature on protocol biopsies in pediatric kidney recipients is scarce and future (randomized controlled) trials or small group trials are needed to address significance of early subclinical rejection and therapeutic interventions.

Besides, possible benefits of protocol biopsies should be weighed against the potential risks such as arteriovenous fistulas and bleeding (207, 208). Because of the improved immunosuppressive regimens, rejection rates have decreased and protocol biopsies might be considered as disproportionate. Additionally, there is no consensus on timing and frequency for protocol biopsies (204, 209, 210).

Although new randomized controlled trials or dedicated small group trials could provide valuable insights in this debate, future research should also focus on developing non-invasive methods for detection of subclinical rejection.

#### Anti-rejection Therapy

Acute rejection of the kidney graft can be divided in either anti-body mediated rejection and T-cell mediated rejection (211). Therefore the treatment of rejection depends on accurate diagnosis and graft biopsy remains the gold standard. The Banff-classification is the international consensus method for the description of biopsies (212). However, in case of high clinical suspicion on rejection (within 6 months after transplantation, after reduction in immunosuppressive therapy and rapidly rising creatinine levels) one could consider treatment without a biopsy.

There are several strategies in the treatment of T-cell mediated rejection. Traditionally intravenous pulses of methylprednisolone are used. Other options are polyclonal antibodies such as ATG, monoclonal antibodies against lymphocyte receptors such as alemtuzumab and rituximab and a proteasome inhibitor such as Bortezomib (213).

Nowadays, the immediate use of polyclonal antibodies instead of methylprednisone is debatable. There is currently little evidence favoring one specific strategy and clinical decision making remains challenging.

There are various possible strategies to treat anti-body mediated rejection, however the optimal therapy remains controversial. Some of the treatment options are similar as in T-cell mediated rejection such as polyclonal antibodies and methylprednisolone. Other possibilities are plasma-exchange, administration of intravenous immunoglobulins (IVIG) and the monoclonal antibody rituximab (214).

In the past, plasmapheresis was common practice whereas this has a less prominent place in anti-rejection therapy nowadays. Billing at al. introduced the combination of IVIG and rituximab which reduced donor specific antibodies and stabilized renal function (215). Although a variety of studies was conducted on anti-body mediated rejection, they used diverse end-points which makes comparisons difficult (213).

Apart from anti-rejection therapy, changes in maintenance therapy should be considered (216).

##### Radboudumc Amalia Children's Hospital

In our center, graft biopsies are performed on clinical indications such as a deterioration of GFR. Five percent of our latest 100 pediatric kidney recipients had a graft biopsy during their transplantation admission. During a median follow-up of 47 months, 42 patients had at least one graft biopsy that mostly revealed calcineurin toxicity (32%) and acute rejection (30%). In total, 36 patients received pulsatile methyl prednisone during follow-up. We have reported one case of life-threatening respiratory failure after alemtuzumab administration (217).

### Medication

#### Immunosuppressive Regimens

##### Historical Developments

One of the factors that improved graft survival is the substantial change in immunosuppressive strategies over time.

In the early days of pediatric kidney transplantation, immunosuppression consisted of total body irradiation and splenectomy which resulted in infection related mortality up to 72% (3, 4). This method was abandoned when corticosteroids were introduced in 1960. As a result, rejection rates increased to 85%. Consequently, the search for better protocols continued with the ultimate goal to minimize severe infections, organ rejections and prevent side effects. Novel immunosuppressive agents, and incorporation of newer prophylactic strategies contributes in achieving this holy grail hopefully in the near future.

In the late 60's more potent medication were available like 6-mercaptopurine and **azathioprine** (antimetabolites). After the introduction of calcineurin inhibitor (CNI) **cyclosporine** in the 1980's, graft survival increased substantially (4, 218, 219). In the past decades, various types of immunosuppressive drugs became accessible.

In the 90's CNI **tacrolimus** and **mycophenolate mofetil** (MMF, a prodrug of mycophenolic acid (MPA), an inhibitor of inosine-5′-monophosphate dehydrogenase) were introduced and the twenty-first century welcomed mammalian target of rapamycin (mTOR) inhibitors like **sirolimus** and **everolimus** (4, 10).

##### Current Practice

Today, maintenance immunosuppressive protocols combine multiple drugs with various modes of action. In this regime CNI, antimetabolites, mTOR-inhibitors and/or corticosteroids for anti-rejection maintenance prophylaxis are the cornerstone (11).

CNI-withdrawal was found to be deleterious for graft function and survival (18). Tacrolimus seems favorable over cyclosporine since it resulted in less acute rejection and improved graft survival (19). Moreover, tacrolimus has less cosmetic side effects than cyclosporine which might be important regarding medication adherence (220).

Similarly, MMF was shown to be a more potent immunosuppressant compared to azathioprine and therefore first choice in antimetabolites. On the other hand, MMF is known for multiple side effects including gastro-intestinal symptoms and anemia which might compromise medication adherence (221).

Corticosteroids are known for their multiple side effects like growth retardation, osteoporosis, hypertension, diabetes mellitus, obesity, dyslipidemia, impaired wound healing, and mental disorders. Currently, 90% of the immunosuppressive protocols contain corticosteroids, despite the demand for minimization. Several studies showed that late steroid-withdrawal is safe in terms of graft survival and rejection in patients with low immunological risks (222, 223).

Other studies showed that early steroid withdrawal is safe as well (224, 225).

On the other hand, earlier research suggested that steroid withdrawing protocols lead to higher incidences of viral infections and post-transplantation lymphoproliferative disease (PTLD) (79, 226). This was suggested to be caused by high dosages of other immunosuppressants in order to compensate for the loss of corticosteroids. In addition, the use of mycophenolate mofetil instead of glucocorticosteroids, is associated with more frequent and severe leukopenia, anemia, and gastrointestinal disturbances (79).

Although corticosteroid withdrawal seems safe in a selected population, long-term effects should be studied before general implementation.

Currently, there is no worldwide consensus on the use of induction therapy. Current literature showed no advantages in a standard, low-risk pediatric population (223, 224). If used, most common induction regimens are with antilymphocyte biological agents, T-lymphocyte-depleting rabbit-derived antithymocyte globulin (rATG), an IL-2 receptor antagonist (IL2RA) like **Basiliximab** (a chimeric (human/murine) monoclonal antibody) or **Alemtuzumab** (an **anti-CD52 T-cell** and B-cell–depleting monoclonal antibody) (11).

##### Radboudumc Amalia Children's Hospital

**Figure 5:**
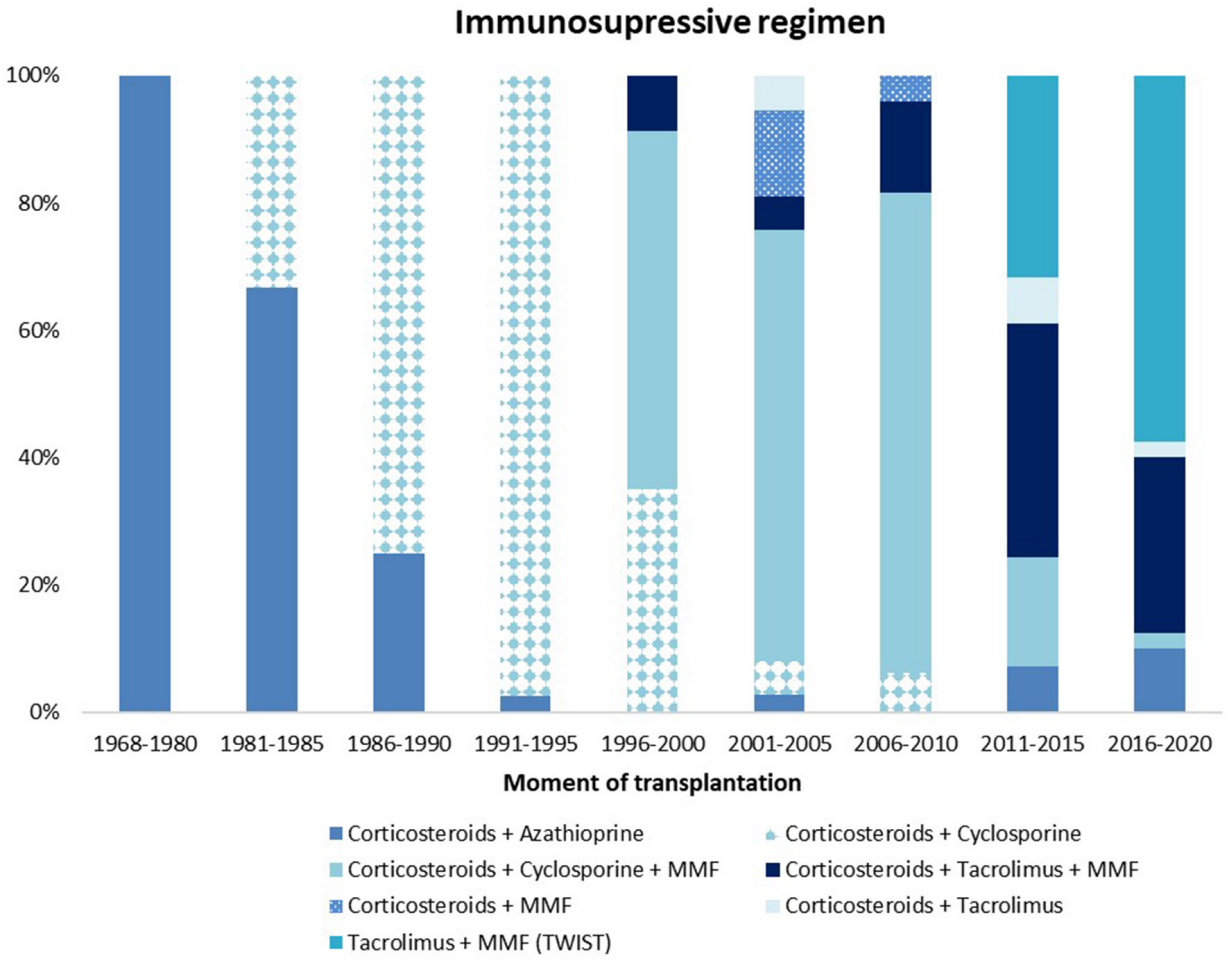
Immunosuppressive maintenance regimen at 3 months after transplantation. MMF, mycophenolate mofetil.

In our center, the use of cyclosporine and prednisone decreased over time, which is comparable to international literature (Figure 5). The Transplantation WIthout Steroids (TWIST) protocol, that limits use of prednisone to 5 days, was introduced in 2012 in recipients without additional risk factors like high sensitization or diseases that are known for their risk on recurrence (224).

Of the most recent 100 recipients, 44 patients started the steroid-sparing TWIST regimen and 44% remained on this regimen during a follow-up period of 5-years. Patients on a steroid-based regimen had significantly more UTIs (63 vs. 25% *p* < 0.01), more CMV infections (11 vs. 0% *p* = 0.03) and more rejections (18 vs. 0% *p* = 0.02) than those on a steroid-sparing regimen. Other side effects did not differ between steroid-sparing and steroid-containing regimens (227). Steroid withdrawal was not associated with improved growth, increased incidence of PTLD, rejection or graft loss in this limited population.

Although the majority of patients started with MMF after transplantation, 45% needed to stop MMF due to side effects, despite the use of slow release Mycophenolate in such cases.

#### Anti-viral Therapy

Children on immunosuppressive therapy are prone to viral infections with potentially severe consequences. Live vaccines cannot be administered after transplantation and inactive vaccines might not be effective due to immunosuppressive agents (228). Therefore, vaccination status is an important issue to optimize before transplantation.

Viral infections that commonly cause morbidity in pediatric kidney recipients are Cytomegalovirus (CMV), Epstein-Barr virus (EBV) and BK-virus (BKV). The latter specifically harms the graft.

Without prophylaxis, these infections are most likely to occur in the early months after transplantation because of transmission trough the graft and high dosages immunosuppression during this period (9, 229).

The use of CMV prophylaxis is recommended in several guidelines, however which patients should receive prophylaxis and for how long remains debatable (229, 230). Valganciclovir is commonly used as prophylactic oral drug whereas ganciclovir is used for therapy.

##### Radboudumc Amalia Children's Hospital

In our center, high risk patients (D+R-) receive CMV prophylaxis until 3 months after transplantation. In the 100 most recent transplanted patients, 62% used CMV prophylaxis. During a median follow-up of 47 months 14% developed CMV disease regardless of prophylaxis.

#### Medication Adherence

Medication adherence is commonly defined as when the patient follows recommendations and instructions from health care professionals concerning the taking of medication that were previously agreed on (231).

Medication non-adherence (MNA) to immunosuppressant regimens is an important factor limiting graft survival. Moreover, in adolescents (aged 11–21 years) it is considered the most important modifiable risk factor for graft loss (232, 233). The risk of acute rejection is doubled when patients are non-adherent whereas the risk of graft loss increases with 80% (234).

The overall prevalence rate of MNA is considered between 30 and 50%, however numbers vary according to the characteristics of the patient population, definition of MNA used, timing of measurements and methods used to assess adherence (233, 235, 236). Despite different percentages mentioned in the literature, it is well-established that adherence is worse in adolescents than in younger children (233, 235, 237).

##### Measurement of MNA

Previous studies reported a wide range of assessment methods like pill counts, questionnaires, patients' diaries, and random measurements of blood drug concentrations. Despite the several methods available, it remains difficult to assess MNA because all methods have their own limitations (238). In general, health care providers underestimate MNA (239).

Despite its limitations, electronic monitoring is currently accepted as the most reliable measurement of adherence (240). In adult population, electronic monitoring results were directly associated with clinical outcome (241).

##### Risks for MNA

The WHO has classified the factors of MNA in 4 categories: individual level, family level, health-care system, and community level (242). Most studies focused on one or few determinants whereas multidimensional assessment might be desirable. Moreover, risk factors vary across countries, types of health care and ethnicities which makes it difficult to draw general conclusions.

At the individual level, recipient age was found to be one of the strongest factors affecting the risk for MNA (235, 243). Qualitative studies assessing barriers to adherence showed that in adolescents one of the main challenges was remembering to take medication, especially on days when there was no strict routine (like weekends and holidays) (232).

Transition of responsibility remains a difficult topic in this age category as these teenagers often desire more independence (244). In a Dutch study on transition, immigrant patients appeared to be particularly at risk for acute rejection during this period (245).

##### Improving MNA

Although many adherence-promoting methods were developed, single strategies were not shown to be effective (246, 247). Previous researchers suggested multi-component behavioral interventions to aim at multiple barriers to adherence (232, 248).

Although some trials with these multi-component interventions have been conducted successfully, these were labor-intensive and not easy to incorporate in daily life (232, 248, 249).

Studies with electronic pillboxes and eHealth interventions have shown promising results in studies among patients with chronic diseases. However, these should be tailor made for this specific population (233, 250–252). On the other side, concerns were raised about these interventions regarding privacy regulations and the large volumes of data health care providers have to deal with in limited time (232).

In addition, special attention should be drawn to the transition of recipients with delayed development. Since they will need extra support, they are prone to fall between two specialties due to their individual transition requirements, especially if they have comorbidities. Taken together, these results suggest that simple “one size fits all” interventions are not effective and that future interventions should be multidimensional and targeting risk levels on various levels in the healthcare system.

##### Radboudumc Amalia Children's Hospital

In our center, medication adherence is addressed every outpatient visit, especially in adolescents. To support transition to adult care, patients are actively prepared from the age of 12 years in a personalized manner. Aspects of this transition phase are education on their disease, the different drugs, outpatient clinic visits with the first part of the visit without their parents. These patients are guided to take responsibility for their treatment. However, actual data on adherence are missing. Little is known on the non-adherence rates in our center.

## Discussion

In the past decades, innovations in pediatric kidney transplantation led to increased graft and patient survival. Despite these innovations, we endeavor to optimize clinical care for pediatric kidney recipients. In this article we provided a state of the art overview and identified paucity of evidence on several important issues in comparison with our results. Future possibilities to improve pre-, peri-, and post-operative care are discussed below.

### Pre-operative Factors

Choosing between an old well-matched living donor and young poorly-matched deceased donor requires consideration of multiple aspects such as sensitization, existing waiting list time and the risk of graft failure (253). Future research should focus on combining these elements in order to make an appropriate trade off and achieve tailor made treatment. The development of validated algorithms would assist clinicians in their considerations for a suitable donor. Thresholds in developing such models include the relatively small numbers of pediatric kidney transplantations and the many changes in practice over time.

In order to fill in the knowledge gaps mentioned above, thorough and well-designed studies are needed. Previous literature is mainly based on retrospective studies in relatively small cohorts. Since the incidence of graft loss has impressively decreased, large volumes are needed to draw conclusions on the factors leading to graft loss.

Besides, research in such a rapid changing field is challenging since data are quickly outdated and confounding factors such as innovations in practice are difficult to correct for.

A possible solution for these problems might be collaboration between the different (inter)national registries. Currently, multiple organizations and registries are actively studying pediatric kidney transplantation such as the NOTR (national) and CERTAIN registry (international). However, each registry collects different data in a slightly different population. Collaboration between those registries allows studying large volumes of data and comparison of practices among different countries. A promising phenomenon is the development of European Reference Networks (ERN), which allows cooperation at the European level between clinicians with specialized expertise. They aim to improve diagnoses and treatment for patients with rare diseases and/or complex conditions. The ERN eUROGEN aims to improve diagnosis, create more equitable access to high-quality treatment and care for patients with rare uro-recto-genital diseases and complex conditions needing highly specialized surgery (254). ERKNet does the same for patients with rare kidney diseases.

### Peri-operative Factors

Surgical techniques have improved over time which resulted a reduction of peri-operative complications. Transplantation in small children is possible although blood pressure and perfusion are vulnerable. Peri-operative monitoring enables strict regulation of those parameters and a multidisciplinary team working according well-defined protocols is mandatory (13, 14, 76).

Although in adults, a refluxing ureteroneocystostomy was shown to be comparable to anti-refluxing methods, less is known about the pediatric population. To reveal this topic future multicentered research should focus on differences in long-term outcome regarding graft function, UTIs and urological interventions. Thresholds for such studies are the confounding factors that differ between centers and the lack of routine VCUG to determine the rate of (asymptomatic) VUR.

### Post-operative Factors

Immunosuppressive protocols have dramatically changed over time. The withdrawal of steroids was shown to be safe in low-risk patients regarding both graft survival and side effects. This remains uncertain for high-risk patients and long term outcome needs to be established.

It remains challenging to find the best combination of immunosuppressive agents, as the balance between preventing rejection while limiting side effects is precarious. Future research should focus on the long term effects of immunosuppressive medication, especially regarding long term side effects. Biotechnical advancements might result in withdrawal of conventional immunosuppression. Current studies focus on cell-based therapy, which aims at the induction of donor-specific unresponsiveness in the setting of either operational tolerance or mixed chimerism (255).

Medication non-adherence has been increasingly recognized as a cause of graft failure, especially in children and adolescents (12, 231). A better understanding of non-adherence is needed and current literature advocates to tailor interventions to each transplant recipients' unique needs, motivations, and barriers. However, previous studies mainly focused on patient- and family factors, the influence of health care providers and health systems are still to be determined. Future research should incorporate the pitfalls for clinicians and health systems in order to optimize medication adherence.

### Future Perspective

Nowadays, artificial intelligence (AI) plays a major role in daily life. Application of AI in medicine and research is becoming more common, especially regarding risk assessment. Machine learning techniques are shown to be promising in processing biomedical data where they are successful for predictive models, image processing and genomic data analysis (256).

In adults, AI has been successfully implemented in the field of kidney diseases. Kuo et al. (257). designed an application that automatically estimates glomerular filtration rate using ultrasound images and multiple studies have shown the benefits of automatic analysis of histological or radiological images (258, 259).

However, such models should be based on a representative cohort of patients, which is a problem in pediatric kidney transplantation. Because of the small patient volumes, development of reliable algorithms and proper validation remains challenging. Although many countries do have their own databases, international collaboration is needed as well as standardization of data, identification of the patient and linking between the different registries (256).

Whereas, optimization of current care remains of utmost importance, recent technologies might offer new perspectives to renal replacement therapy. Current research focusses on developing a wearable artificial kidney that would improve both quality of life and quality of dialysis (260, 261).

Another promising field is that of regenerative medicine, the process of generating a human kidney *de novo* has been studied over the last decades (262–265). Several authors used pluripotent stem cells to form kidney precursors cells and eventually organoids (266, 267). Differentiation is shown to be limited in 3D cultures and those cultured organoids lack several important structures such as the loops of Henle. However, when placed into living animals these organoids develop capillary loops and connect to the hosts' vascularization. Nowadays, tissue derived from pluripotent stem cells is used to study the genetics aspects of kidney diseases (262, 268).

Despite these promising developments, there are many hurdles to take before one could generate a functioning human kidney. Among them are the possible tumorgenicity, the small scale of organoids and absence of potent vascularization and urinary drainage system (262, 269, 270). Therefore, using newly grown human kidney tissue for renal replacement therapy is still some time off.

Another solution for the graft shortage might be xenotransplantation with the kidney of genetically modified pigs. Recently, surgeons have placed such a kidney in a brain-dead patient for research sake (271). Both the kidney and the thymus were transplanted with good outcome in the first 54 h after transplantation. These findings have not been peer-reviewed and published yet and the procedure will not be available to patients any time soon. Both medical and ethical objections need to be considered first and long terms effects need to be studied.

## Conclusion

This overview of 50 years care for pediatric kidney recipients revealed an impressive improvement of graft and patient survival. Important developments are the increased use of living donors, improved immunosuppressive therapy and better peri-operative care.

Still, many questions remain unanswered. In our center, pre-transplant treatment modality, donor age and HLA mismatching did not affect graft survival which might advocate donor pool expansion. More large scale, multicenter studies are needed to confirm these findings.

Since urological complications are more common in children, an active screening program for LUTD should be considered. Moreover, the optimal method for surgical vesico-ureteral anastomosis still needs to be established.

## Data Availability Statement

The raw data supporting the conclusions of this article will be made available by the authors, without undue reservation.

## Author Contributions

LO: collected the data, performed the analysis, and wrote the paper. CB-R and LW: conceived and designed analysis, collected the data, and provided intellectual guidance. EC: collected the data and provided intellectual guidance. WdF: conceptualization, writing—review and editing, and supervision. All authors contributed to the article and approved the submitted version.

## Conflict of Interest

The authors declare that the research was conducted in the absence of any commercial or financial relationships that could be construed as a potential conflict of interest.

## Publisher's Note

All claims expressed in this article are solely those of the authors and do not necessarily represent those of their affiliated organizations, or those of the publisher, the editors and the reviewers. Any product that may be evaluated in this article, or claim that may be made by its manufacturer, is not guaranteed or endorsed by the publisher.
