# Supplementary material for: Pearls and Pitfalls in Pediatric Kidney Transplantation After 5 Decades
Source: Front Pediatr. 2022 Apr 8;10:856630. doi: 10.3389/fped.2022.856630 (PMC9024248; doi:10.3389/fped.2022.856630)
Supplement: Supplementary file 1 [file Data_Sheet_1.docx]

**Appendix**

**Analysis Radboudumc data**

Data collection started at moment of transplantation until graft loss or death with functioning graft. Extensive recipient-, donor-, and transplantation-related data were collected e.g. donor and recipient age, cause of renal failure, year of transplantation, LD or DD, cold and warm ischemia time, Human Leukocyte Antigen (HLA) mismatches, graft loss and graft function over time.

Primary outcome was graft loss over time, graft loss was defined as the start of any other form of renal replacement therapy.

**Statistical analysis**

Categorical data were described with percentages. Continuous data were described using mean and SD for normally distributed data and median and range for non-normally distributed data (tested with Mann-Whitney analyses). All numerical variable were tested for normal distributions using the Kolmogorov-Smirnov normality test

Graft survival was analyzed using Kaplan-Maier curves. Differences in survival was checked for significance with log-rank tests.

Finally, a prognostic backward multivariate analysis was calculated using Cox regression in order to analyze the influence of donor age on graft survival (primary outcome variable). Confounding variables included were age difference between donor and recipient, donor type, number of total HLA mismatches, number of HLA-DR mismatches, warm ischemia time, cold ischemia time, underlying disease causing ESKD, pre-transplantation treatment, number of previous transplantations and occurrence of delayed graft function. The model was corrected for era of transplantation (per decade).


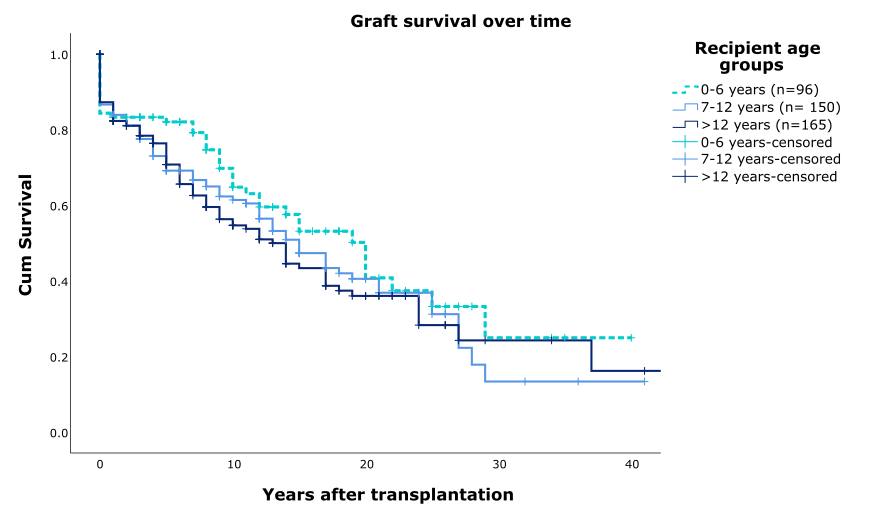


**Supplementary Figure 1. Graft survival by recipient age.** p=0.0260
